# Pandemic Lessons From a Long-Term Care Ombudsman

**DOI:** 10.1093/geroni/igab046.1502

**Published:** 2021-12-17

**Authors:** Mairead Painter

**Affiliations:** Long-Term Care Ombudsman Program, Hartford, Connecticut, United States

## Abstract

This session will provide updates on how the pandemic led to horrific situations in long-term care facilities and how the pandemic influenced major federal efforts to address elder abuse, neglect, and exploitation.

